# Differences in the effect of repetitive transcranial magnetic stimulation and median nerve electrical stimulation in patients with prolonged disorders of consciousness after intracerebral hemorrhage: a randomized controlled trial protocol

**DOI:** 10.3389/fneur.2024.1511767

**Published:** 2024-11-28

**Authors:** Guoshun Zhao, Mingzhu Fang, Shiyu Han, Xiaoke Peng, Anqin Dong

**Affiliations:** Department of Rehabilitation Medicine, The Fifth Affiliated Hospital of Zhengzhou University, Zhengzhou University, Zhengzhou, China

**Keywords:** prolonged disorders of consciousness, median nerve electrical stimulation, repetitive transcranial magnetic stimulation, EEG, functional near infrared spectroscopy

## Abstract

**Background:**

Repetitive transcranial magnetic stimulation (rTMS) and median nerve electrical stimulation (MNES) are two non-invasive neuromodulation techniques that have demonstrated potential in facilitating the recovery of consciousness in patients with impaired consciousness. However, existing studies on awakening interventions for patients with prolonged disorders of consciousness (pDoC) following intracerebral hemorrhage remains limited. In particular, systematic comparisons of the efficacy of rTMS versus MNES in this specific patient population are lacking.

**Methods:**

This is a single-center randomized controlled trial in which 45 patients will be randomly assigned to the control group, rTMS group and MNES group. The intervention period will lasts 4 weeks. All patients underwent multimodal assessments before and at the end of treatment, which were used to comprehensively evaluate their recovery of consciousness and changes in brain function. The assessments includes the Coma Recovery Scale, electroencephalogram, event-related potentials (P300 and mismatched negative) and functional near-infrared spectroscopy.

**Discussion:**

This study represents the first systematic comparison of the efficacy between rTMS and MNES in patients with pDoC following intracerebral hemorrhage. The objective is to employ multimodal assessment techniques to provide clinical references into the individualized application of these neuromodulation therapies.

**Clinical trial registration:**

https://www.chictr.org.cn/, identifier ChiCTR2400082022.

## Introduction

Prolonged disorders of consciousness (pDoC) encompass a spectrum of conditions caused by severe brain injury, including unresponsive wakefulness syndrome and minimally conscious state (1). These patients are in a prolonged state of absent or severely limited consciousness, which seriously affects their quality of life (2). Due to their inability to live independently, they impose a huge medical and economic burden on society (3). Most of the existing studies have focused on traumatic brain injury or have not specified the etiology, while relatively few studies have addressed pDoC resulting from intracerebral hemorrhage (4). pDoC after intracerebral hemorrhage often carry a poor prognosis (5), and it highlights the need for research and treatment development for this specific etiology (6).

Non-invasive neuromodulation techniques have shown some potential in improving the prognosis of pDoC patients. Repetitive transcranial magnetic stimulation (rTMS) works by generating rapidly changing magnetic fields in specific regions of the cerebral cortex, which induce neuronal depolarization. This process modulates neuronal excitability and synaptic plasticity, aiming to promote arousal in patients with pDoC (7). Wan et al. (2) showed that the application of high-frequency rTMS significantly improved coma recovery scale-revised (CRS-R) scores, and provided further evidence through event-related potentials (ERPs). Bai et al. (8) confirmed that rTMS improved cortical excitability and cortical connectivity in pDoC patients by electroencephalogram (EEG) and TMS-EEG, explaining the potential mechanism of rTMS in pDoC wakefulness promotion.

Median nerve electrical stimulation (MNES) is a peripheral neuromodulation technique, which activates the ascending reticular activating system (ARAS) by enhancing sensory afferents through direct stimulation of the median nerve (9). In turn, it improves the neural activity and functional connectivity of the frontal lobe and other cortical areas related to consciousness, and promotes the recovery of consciousness (10). Most studies on MNES in promoting awakening have focused on coma patients (11). Xiong et al. (12) reported that MNES significantly enhanced the cortical brain activity with pDoC, and this effect was confirmed through the EEG. It suggests the potential value of MNES in boosting consciousness levels in pDoC.

Functional near-infrared spectroscopy (fNIRS) is a non-invasive brain imaging technique that has gained traction in recent years. Compared to other imaging techniques such as functional magnetic resonance imaging and positron emission tomography, fNIRS exhibits significant advantages in a number of ways. Its high temporal resolution allows for rapid acquisition of cortical oxygenation data (13). fNIRS is mobile and flexible enough to be used at the bedside or in dynamic environments. In addition, fNIRS is less affected by motion artifacts and still obtain more accurate brain data with patients’ slight movements (14). Si et al. (15) demonstrated that fNIRS was able to effectively differentiate changes in cortical blood oxygenation in different states of consciousness, validating the potential application of fNIRS in pDoC studies.

This study aims to systematically compare the efficacy of rTMS and MNES in pDoC patients after intracerebral hemorrhage by multimodal assessments. This research offers new perspectives on the distinct mechanisms of central and peripheral neuromodulation techniques and their clinical applications. The findings are expected to inform the optimization of future treatment plans and the development of personalized intervention strategies.

## Methods

### Study design

**Figure 1:**
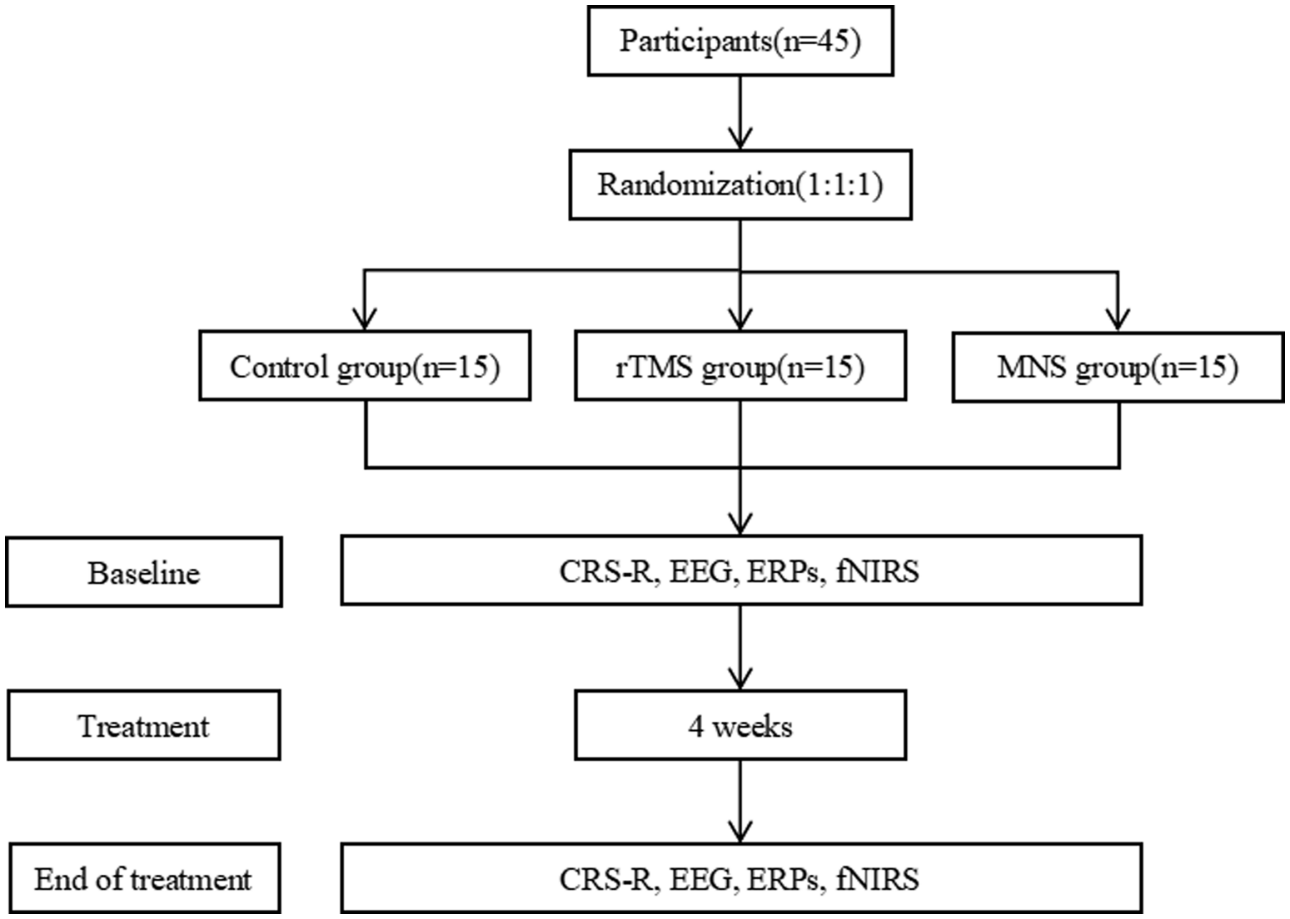
The study design. rTMS repetitive transcranial magnetic stimulation; MNS, median nerve electrical stimulation; CRS-R, coma recovery scale-revised; ERPs, event-related potentials; FNIRS, functional near-infrared spectroscopy.

This study is a single-center, randomized controlled trial (Figure 1). The study complies with the Declaration of Helsinki and was approved by the Ethics Committee of the Fifth Affiliated Hospital of Zhengzhou University (Approval No. KY2023093).

### Participants

All patients will be recruited from the Department of Rehabilitation Medicine, The Fifth Affiliated Hospital of Zhengzhou University, China. The study lasts from December 2023 to December 2024.

Inclusion criteria: (1) Diagnosed with hemorrhagic stroke, and assessed as minimally conscious state (MCS) or unresponsive wakefulness syndrome (UWS) according to the CRS-R assessment (16); (2) Aged between 18 and 75 years; (3) Normal brainstem auditory evoked potentials on at least one side; (4) No history of craniotomy involving the left dorsolateral prefrontal cortex; (5) The guardian signed an informed consent.

Exclusion criteria: (1) Intracranial metal implants or large cranial defects; (2) Unstable vital signs; (3) Seizures within the past 4 weeks; (4) Concurrently receiving other neuromodulatory treatments; and (5) Participants who withdrew from the study midway.

### Sample size

Sample size estimation was estimated using G-Power software. The CRS-R effect size was based on similar studies from the literature (17). The effect size was taken as 0.62, *α* = 0.05, and efficacy power (1-*β*) = 0.95. Considering a dropout rate of 20%, the total sample size was calculated to be 45 cases. The results of the sample size calculation provided a solid foundation to ensure that the effect of the intervention could be effectively detected in this study.

### Randomization and blinding

The randomized sequence will be generated by SPSS 26.0 software. Patients will be divided into 3 groups according to the order of enrollment in a 1:1:1 manner: 15 cases in the conventional group, 15 cases in the rTMS group, and 15 cases in the MNES group. The results of the patient grouping will be sealed in opaque envelopes to ensure concealment of the allocation, and these envelopes will be kept by a special person and revealed only at the time of allocation. For objectivity of the study, patients, assessors and data analysts will all be blinded. It ensures that potential bias is avoided during evaluation and data processing.

### Treatment

All participants will receive conventional wakefulness-promoting treatments recommended by specialized physicians, including medications, hyperbaric oxygen, Chinese acupuncture and exercise therapy. These conventional treatments aims to provide comprehensive support for patients, promote the recovery of neurological function, and establish a foundation for subsequent neuromodulation interventions. In addition, patients in the control group will receive sham rTMS and MNES interventions. Patients in the rTMS group will receive sham MNES interventions, and patients in the MNES group will receive sham rTMS interventions. In addition, the number of cases of adverse reactions such as seizures, scalp burns, and myogenic spasms in patients will be recorded during the experiment.

### rTMS treatment

The target of rTMS stimulation is the left dorsolateral prefrontal cortex (L-dLPFC). The stimulation frequency is 10 Hz and the stimulation intensity is 90% of the resting motor threshold (RMT). Each session consistes of 1,000 pulses, delivered in 10 sets of 100 pulses each, with a 60-s interval between sets. The total stimulation time per session is 10 min and 40 s. Treatment is administered 6 times per week for 4 weeks.

rTMS treatment is administered using a magnetic stimulator (YRD-CCY-II, Yiruide, China) with a circular coil of 12.5 cm in diameter and a peak stimulation intensity of 3 T. RMT measurement is first performed. Patients are positioned in a supine posture, ensuring full-body relaxation. Recording electrodes are placed on the belly of the right abductor pollicis brevis muscle, with a reference electrode attached. TMS is applied near the left M1 region, and motor evoked potentials (MEPs) is recorded. Stimulation intensity is gradually increased from a low level until stable MEP is elicited. Then the intensity is gradually reduced until the minimum level at which at least 5 out of 10 consecutive stimulations produced MEP with an amplitude of ≥50 μV in the right abductor pollicis brevis. This value is defined as rMT. Following the 10–20 International EEG System, the coil center is positioned over the L-dLPFC (near F3), ensuring close contact with the scalp to guarantee effective stimulation transmission.

### Sham rTMS treatment

The sham rTMS group uses the same device as the rTMS group, but with the coil positioned with its back facing the scalp. This setup does not generate an effective electromagnetic field to target the cortex, though the device still produces slight vibrations and sounds during operation. As the patients are unconscious, blinding is effectively maintained throughout the intervention. The frequency, intensity, pulse count, and duration of the sham rTMS matched those of the active rTMS group.

### MNES treatment

A portable multiparameter monitor (X5-ER, Nuocheng Electric, China) is used for MNES treatment. The patient is placed in the supine position. The right forearm is placed flat to accurately localize the median nerve position. Before treatment, the skin is sterilized using alcohol cotton balls to ensure adequate contact between the electrode and the skin. The anodic electrode is placed 2 cm above the transverse carpal stripe on the palmar side of the right wrist joint, while the cathodic electrode is attached to the right interosseous muscle. The current amplitude is 20 mA, frequency 40 Hz, pulse width 300 μs. Each stimulation lasts 20 s, followed by a 40-s interval. 30 min of stimulation per time, twice a day, 6 d/w, for 4 weeks. The entire treatment process is monitored by a professional to ensure safety and maximize the therapeutic effect.

### Sham MNES treatment

The sham MNES group uses the same equipment as the MNES group, but does not activate the electrical stimulation button. The patient still goes through the same electrode attachment and equipment preparation process, but does not feel the electrical stimulation.

### Outcomes

#### CRS-R

The CRS-R is one of the most accurate and recommended behavioral scales for the evaluation of consciousness in pDoC. It assesses the state of consciousness from six different perspectives: auditory, visual, motor, verbal, communication, and arousal (18). CRS-R’s unique multi-dimensional design enables it to acutely reflect patients’ responses in different situations. In particular, it shows high sensitivity in distinguishing the difference in level of consciousness between MCS and UWS patients. The evaluator assesses the patient five times within 7 days and selects the highest score to be used for classification. Based on the scores, the patients’ level of consciousness can be categorized as UWS, MCS-, MCS+, and emergence from MCS (19).

#### EEG

Resting-state EEG is assessed using a 32-channel brain function monitor (Natus, USA). The leads are placed according to the 10–20 International EEG system to ensure coverage of the patient’s cortical areas. According to Hockaday’s grading criteria, EEG is categorized into grades I-V to differentiate between different degrees of cerebral functions (20). Grade I: Normal, regular alpha waves with few theta waves and reactivity; Grade II: Mildly abnormal, characterized predominantly theta waves; Grade III: Moderately abnormal, predominantly delta waves or spindle coma; Grade IV: Severely abnormal, showing burst-inhibition or alpha coma or delta coma or predominantly delta waves amplitude of <20 μV; Grade V: extremely abnormal, almost flat waves or no EEG activity (<2 μV).

#### P300

The P300 is recorded using the Oddball paradigm with a brain function monitor (Natus, USA). Patients receive auditory stimulation in a quiet environment. The experiment includes a total of 300 stimuli, consisting of 80% standard tones (1,000 Hz, 100 ms) and 20% target tones (1,500 Hz, 100 ms), with a tone interval of 1.5 s (21). The EEG signals are recorded through Cz electrodes with a sampling rate of 500 Hz. The filtering range is set to 0.1–30 Hz, and the reference electrodes are bilateral earlobes (A1 and A2).

#### Mismatch negativity (MMN)

MMN is recorded by differential auditory stimulation sequences using a Brain Function Monitor (Natus, USA). Patients lay flat on a hospital bed in a quiet environment without active responses. A total of 1,000 stimuli are provided during the experiment, 80% of which are standard tones (1,000 Hz, 100 ms) and 20% deviant tones (1,200 Hz, 100 ms), with a tone interval of 1.5 s (22). EEG signals are recorded through Fz electrodes. The sampling rate is 500 Hz, and the filtering range is 0.1–30 Hz. The reference electrodes are placed on the bilateral earlobes (A1, A2).

#### fNIRS

**Figure 2:**
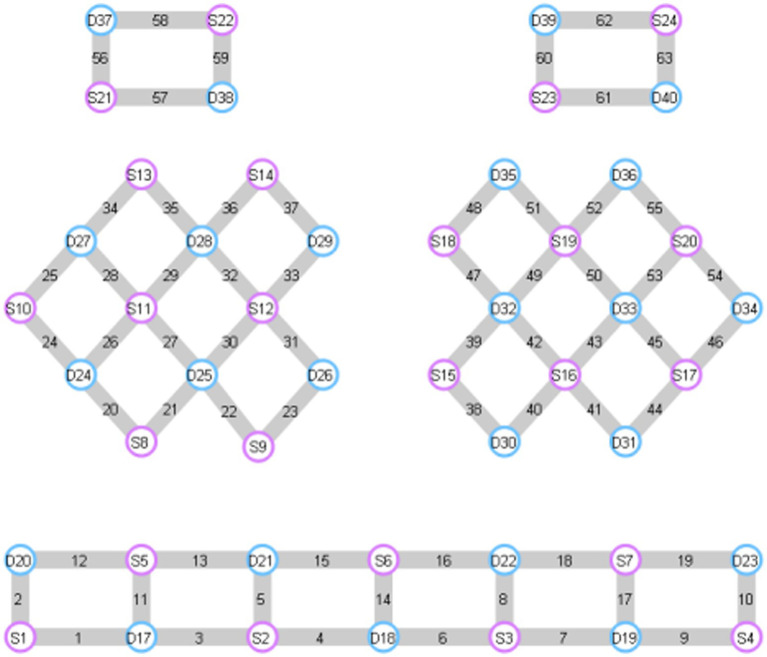
FNIRS channel diagram. The red circles represent the light source, the blue circles represent the detectionse, and the gray parts are the formed channels. L-DLPFC: 16, 17, 18, 40, 41. R-DLPFC: 11, 13, 15, 20, 21. L-PMC: 38, 39, 42, 43, 45. R-PMC: 23, 26, 27, 30, 31. L-M1: 47, 49. R-M1: 32, 33. L-S1: 50, 53, 54. R-S1: 25, 28, 29. L-PFC: 6, 7, 8, 9, 10, 16, 17, 18, 19, 40, 41, 44. R-PFC: 1, 2, 3, 4, 5, 11, 12, 13, 15, 20, 21, 22.

fNIRS data acquisition is performed using NirScan-6000A experiment (Danyang Huichuang Medical Equipment Co., Ltd., China). The sampling rate is 11 Hz and wavelengths are 730 nm, 808 nm and 850 nm. The experiment uses 24 light sources and 40 detectors to form 63 effective channels, the average distance between the source and the detector is 3 cm (range 2.7–3.3 cm), with reference to the international 10/20 system for positioning (Figure 2). Based on previous studies, the regions of interest (ROI) include DLPFC, pre-motor cortex (PMC), primary motor cortex (M1), primary somatosensory cortex (S1) and prefrontal cortex (PFC). The corresponding channels are L-DLPFC (channels 16, 17, 18, 40, 41), R-DLPFC (channels 11, 13, 15, 20, 21), L-PMC (channels 38, 39, 42, 43, 45), R-PMC (channels 23, 26, 27, 30, 31), L-M1 (channels 47, 49), R-M1 (channels 32, 33), L-S1 (channels 50, 53, 54), R-S1 (channels 25, 28, 29), L-PFC (channels 6, 7, 8, 9, 10, 16, 17, 18, 19, 40, 41, 44) and R-PFC (channels 1, 2, 3, 4, 5, 11, 12, 13, 15, 20, 21, 22).

Patients perform a command-driven “hands-on” motor imagery (MI) task. The experimental paradigm consists of an initial rest period (40 s) and five subsequent blocks. Each block consists of a reaction period (30 s) and a rest period (30 s). This task paradigm is particularly suitable for patients with DoC and has been validated for such use (23).

Data processing is performed using MATLAB2024a and HOMER2. First, raw optical density signals are converted into concentration changes of oxyhaemoglobin (HbO) and deoxyhemoglobin (HbR) based on the modified Beer–Lambert law (24). Then, the bandpass filter is 0.01–0.1 Hz and motion artifacts are corrected by spline interpolation (25).

Subsequently, baseline data for each channel is corrected. The average signal of the first 5 s of the response period is used as the baseline. The mean and slope value of the hemodynamic response during the MI task are collected. The mean of the hemodynamic response is extracted from 5 to 25 s after the start of the reaction period. The slope value is extracted from 2 to 7 s after the start of the reaction period (26). The HbO and HbR signals from the ROI are extracted, averaged across channels, used to assess the degree of activation of different brain regions during the MI task.

### Statistical analysis

Data analysis is conducted using SPSS 26.0 software. Normality and homogeneity of variance tests are first performed on the measurement data. Normally distributed data are expressed as mean ± standard deviation. One-way analysis of variance is used for between-group comparisons, and paired t-tests are used for within-group comparisons. For data that do not conform to normal distribution, the median and interquartile range are expressed, and Kruskal-Wallis test is used for between-group comparison. Count data were analyzed for variability by chi-square test. For rank data, Mann–Whitney U test and Wilcoxon signed rank test are used. The confidence interval for all statistical results is 95%, with *p* < 0.05 as the criterion for statistically significant differences.

## Discussion

Neuronal damage resulting from severe intracerebral hemorrhage is not confined to the directly affected area but extends to broader brain networks dysfunction, which leads to different degrees of decline in the level of consciousness (27). In recent years, both international and domestic studies have been conducted in the field of wakefulness promotion in pDoC, but only amantadine and transcranial direct current stimulation show level II evidence (28). In contrast, the application of rTMS and MNES to pDoC is still in the exploratory stage (29). Further investigation is needed to better understand the subgroup of pDoC after intracerebral hemorrhage and the differences efficacy differences of different treatments in this population. To the best of our knowledge, this study will be the first to report the clinical application of non-invasive neuromodulation techniques in pDoC after intracerebral hemorrhage.

Several studies have demonstrated that rTMS and MNES can improve the level of consciousness in patients with impaired consciousness (30). However, these clinical studies vary in terms of Stimulation parameters, etiology, disease course, and duration of intervention. It cannot yet fully explain their specific efficacy in patients with pDoC after intracerebral hemorrhage. Patients with prolonged impaired consciousness post intracerebral hemorrhage often face limitatins in using rTMS due to factors such as history of craniotomy or intracranial metal implantation. On the other hand, MNES has fewer contraindications and is expected to be an alternative therapy to rTMS. On this basis, this study attempted to explore the application of MNES in pDoC after international hemorrhage and to compare its efficacy with rTMS. On this basis, this study attempts to explore the application of MNES in pDoC after intracerebral hemorrhage and compare the efficacy with rTMS by means of multimodal assessment. It further reveals the differences and complementary roles in the mechanisms of central and peripheral neuromodulation techniques.

In this study, the stimulation target for high-frequency rTMS is L-dLPFC. High-frequency rTMS (≥5 Hz) primarily works by enhancing long-term potentiation, promoting neural plasticity in damaged networks and enhancing functional connectivity between brain regions (31). The L-dLPFC plays a key role in higher cognitive functions (32). Based on the midbrain circuit model, the activation or inhibition of prefrontal cortex is closely linked to the recovery of consciousness in pDoC patients (33). As L-dLPFC plays a central role in regulating consciousness and cognitive functions, its impaired functional network connectivity with thalamus, brainstem and other regions is one of the important mechanisms leading to pDoC (34). High-frequency rTMS not only directly activates neurons in the target cortex, but also has significant advantages in enhancing the function of remote brain regions. In addition, high-frequency rTMS improves synaptic plasticity by regulating the balance of glutamate (35) and gamma-aminobutyric acid (36) and increasing the expression of neurotrophic factors (37). It also regulates the inflammatory response in the brain to promote neural repair (38). This also has an irreplaceable role in alleviating brain damage caused by intracerebral hemorrhage.

The main reason for choosing the right median nerve for electrical stimulation is its functional association with the left cerebral hemisphere. Stimulating the right median nerve can maximally activate the left frontal and parietal regions. These regions are closely associated with consciousness and cognitive functions. Choosing the right side also avoids potential interference with the dominant hand and ensures that the patient’s motor function is not compromised during the rehabilitation process. The wakefulness promotion of MNES is achieved through multifaceted effects on the central nervous system. First, the MNES exerts its effects by strengthening synaptic connections between the median nerve and ARAS. The ARAS is a key neural complex that maintains wakefulness. MNES transmits excitatory signals to the ARAS and further to the inner nucleus of the thalamus layer (39). This process eventually promotes cortical activation. Norepinephrine released from the locus coeruleus in the ARAS further enhances cortical excitability and responsiveness. MNES also acts in enhancing neuroplasticity. It has been shown that MNES can improve the levels of brain-derived neurotrophic factors and orexins and they play an important role in nerve repair and synaptic restoration (12). This suggests that MNES has the potential to promote neural regeneration and functional reorganization. In addition, MNES promotes brain metabolism and functional recovery by increasing cerebral blood flow (39). The enhancement of intracerebral blood flow is crucial for the repair of damaged brain tissue during the long-term rehabilitation. MNES also demonstrates a significant role in improving electrophysiological activity. It can enhance neural cell activity and reduce the inhibitory state of the brain (40). This is reflected not only in electrophysiological indicators, but also in the patient’s behavioral responses and level of consciousness. Combined with the current scientific progress, MNES promotes the awakening and recovery of patients with DoC through multiple mechanisms, such as activating ARAS, regulating neurotrophic factors, increasing cerebral blood flow and improving electroencephalographic activity. Due to the extensive role of the median nerve in regulating the central nervous system, MNES has been referred to as the “gateway” from the peripheral nerves system to the central nervous system (30). The synergistic effect of these mechanisms provides a potentially effective way to promote awakening in patients with pDoC after cerebral hemorrhage, and shows a broad clinical prospect.

A key feature of this study is the use of multimodal neurofunctional assessments to comprehensively quantify and evaluate the effects of rTMS and MNES on promoting wakefulness in pDoC patients from intracerebral hemorrhage. Due to multidimensional approach, CRS-R is sensitive for detecting small changes in consciousness (41). In patients with pDoC, EEG shows increases in theta or delta slow waves and decreases in fast waves (42). The Hockaday’s grading is based on scoring the background activity and categorizing the EEG activity into different grades (43). It provide objective data on the state of the patient’s neurological activity and it is especially important in monitoring the recovery of neurological function. A studies suggested that higher EEG grades, like III, IV, and V, are typically associated with poorer prognosis, while I and II lower grades indicate a better potential (44). Wang et al. (45) showed the prognosis of pDoC patients by EEG grading and verified that the EEG grading was closely related to the long-term recovery. A study by Xiong et al. (12) used the EEG grading to assess the efficacy of rTMS in patients with pDoC, confirmed that the improvement in the consciousness level could be reflected by the EEG grading. In addition, EEG grading also had a close correlation with the CRS-R and the subscales.

P300 and MMN have important value in the diagnosis and prognosis of pDoC after intracerebral hemorrhage. P300 mainly reflects the brain’s ability to perceive external information and the degree of the processing of information. It can be used to assess the cognitive responsiveness of the external stimuli (21). Research suggests that the latency of P300 is a reflection of the speed of nerve conduction and the wave amplitude reflects the cognitive ability. Prolonged latency and reduced wave amplitude are usually linked to cognitive impairment in patients with pDoC. Li et al. (46) used the latency and wave amplitude of P300 for prognostic assessment of DoC, showing high sensitivity and specificity. This study also found that P300 correlates with CRS-R. On the other hand, MMN is able to reflect underlying conscious functioning by assessing a patient’s ability to perceive deviant stimuli in an automated manner. The presence and fluctuation of MMN correlate with altered states of consciousness in pDoC patients, and changes in amplitude of the wave precede changes in the level of clinical consciousness (47). Zhang et al. (48) found that the microstates of MMN wave amplitude characterization could distinguish and predict different levels of consciousness in pDoC. Both EEG and ERP (P300 and MMN) provided objective quantification of cortical activity, further validating the neurophysiological basis of behavioral changes.

Task-based fNIRS helps monitor real-time changes in blood oxygen levels of the cerebral cortex and differentiate the activation of different brain regions during a task. It provides a neurological reflection of the function from another perspective (49). The study employs the MI task paradigm to explore the relationship between cortical activation and levels of consciousness during MI. The MI task requires participants to imagine hand movements at rest, which triggers cortical activation similar to actual movement, especially in relevant regions such as M1. The task-based fNIRS can assess the level of residual consciousness in pDoC patients. Unlike the protocol of this study, the task used in the study by Si et al. (15) consisted of six questions, where participants were instructed to imagine playing badminton to respond.

Through multimodal assessment, this study provides an important basis for using rTMS and MNES in the individualized treatment of pDoC after intracerebral hemorrhage. Future studies should aim to validate the effects of these interventions in larger clinical trials, and further explore the differences in efficacy between different neuromodulation techniques and their potential for combined application. The exploration of individualized treatment strategies is also an important direction for future research. By optimizing therapeutic parameters, it is expected to provide a more precise and comprehensive intervention plan for patients with pDoC resulting from intracerebral hemorrhage.
